# Supplementary material for: Identifying GPSM Family Members as Potential Biomarkers in Breast Cancer: A Comprehensive Bioinformatics Analysis
Source: Biomedicines. 2021 Sep 3;9(9):1144. doi: 10.3390/biomedicines9091144 (PMC8471503; doi:10.3390/biomedicines9091144)

Supplementary Data

Supplementary Table S1. GPSM2 over-expression in Cancer vs. Normal analyses (Oncomine)

Dataset	Group comparison	Fold change	p value	Number of patients
Curtis Breast (n = 2136)	Medullary Breast Carcinoma vs. Normal	2.484	5.95E-14	32/176
Zhao Breast (n = 64)	Invasive Ductal Breast Carcinoma vs. Normal	2.180	3.62E-8	35/38
Ma Breast 4 (n = 66)	Ductal Breast Carcinoma in Situ Epithelia vs. Normal	4.391	1.04E-4	9/23
	Invasive Ductal Breast Carcinoma Epithelia vs. Normal	4.448	6.64E-4	9/23
TCGA Breast (n = 593)	Invasive Lobular Breast Carcinoma vs. Normal	2.399	1.00E-12	36/97
	Invasive Ductal Breast Carcinoma vs. Normal	2.844	1.12E-30	389/450
	Invasive Breast Carcinoma vs. Normal	2.536	1.31E-18	76/137
	Mixed Lobular and Ductal Breast Carcinoma vs. Normal	2.569	2.92E-4	7/68
Turashvili Breast (n = 30)	Invasive Ductal Breast Carcinoma vs. Normal	2.347	0.006	5/25
Richardson Breast 2 (n = 47)	Ductal Breast Carcinoma vs. Normal	3.565	5.05E-7	40/47

Supplementary Table S2. Statistics of GPSMs transcript levels between subgroups of individuals with breast cancer (UALCAN). P values in bold are statistically significant (< 0.05).

COMPARISON	P VALUE			
	GPSM1	GPSM2	GPSM3	GPSM4
Sample types				
Normal - Primary tumor	**1.82E-12**	**1.62E-12**	**1.04E-10**	**1.62E-12**
Cancer stages				
Normal - Stage 1	**1.94E-05**	**9.99E-16**	**1.97E-08**	**8.38E-12**
Normal - Stage 2	**3.28E-13**	**1.62E-12**	**3.38E-08**	**1.01E-11**
Normal - Stage 3	**8.85E-08**	**<1E-12**	**6.73E-07**	**2.05E-10**
Normal - Stage 4	**1.21E-02**	**7.54E-05**	4.51E-01	1.49E-01
Stage 1 - Stage 2	1.07E-01	**3.55E-02**	6.99E-02	8.12E-01
Stage 1 - Stage 3	4.40E-01	7.46E-01	1.16E-01	4.71E-01
Stage 1 - Stage 4	8.24E-01	9.95E-02	2.04E-01	2.29E-01
Stage 2 - Stage 3	4.84E-01	**9.20E-04**	8.82E-01	3.48E-01
Stage 2 - Stage 4	2.38E-01	**8.03E-04**	5.2E-01	1.32E-01
Stage 3 - Stage 4	4.61E-01	1.09E-01	4.32E-01	4.15E-01
Breast cancer subclasses				
Normal - Luminal	**8.16E-09**	**1.11E-16**	**8.74E-10**	**2.22E-16**
Normal - HER2 positive	1.32E-01	**3.19E-06**	2.59E-01	**6.54E-10**
Normal - Triple negative	**3.69E-08**	**1.62E-12**	**3.35E-06**	**9.96E-09**
Luminal - HER2 positive	5.87E-01	8.8E-02	2.04E-01	**1.62E-12**
Luminal - Triple negative	**1.10E-02**	**1.62E-12**	1.74E-01	**<1E-12**
HER2 positive - Triple negative	8.24E-02	**1.86E-05**	7.96E-02	4.26E-01

Supplementary Figure S1. mRNA expression levels of GPSMs in post-mortem normal tissues from the GTEx dataset (Human Protein Atlas). RNA-sequencing tissue data are represented as mean pTPM, corresponding to mean values of individual samples from each tissue. *GTEX, Genotype-Tissue Expression; pTPM, protein-coding transcripts per million.*

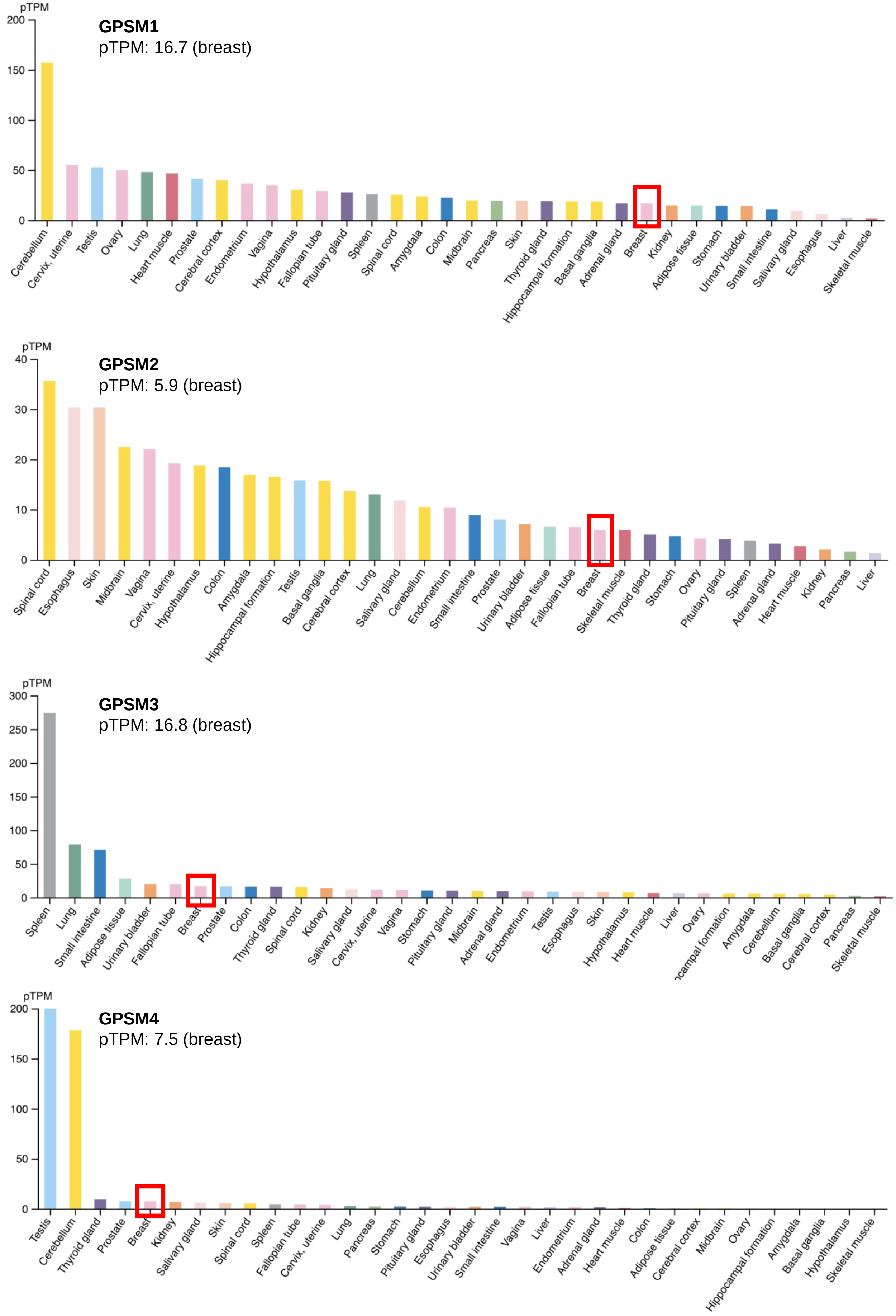

Supplementary Figure S2. Distant metastasis-free survival (DFMS) analysis of GPSM1, GPSM3 and GPSM4 in breast cancer (Kaplan-Meier plot). Red and black curves represent survival analysis for higher and lower GPSMs mRNA expression levels, respectively. Red and black titles indicate statistically and non-statistically significant survival, respectively. *All, overall survival; ER, estrogen receptor; HER2, human epidermal growth factor receptor 2; PR, progesterone receptor; +, positive; -, negative.*

S2-1) GPSM1

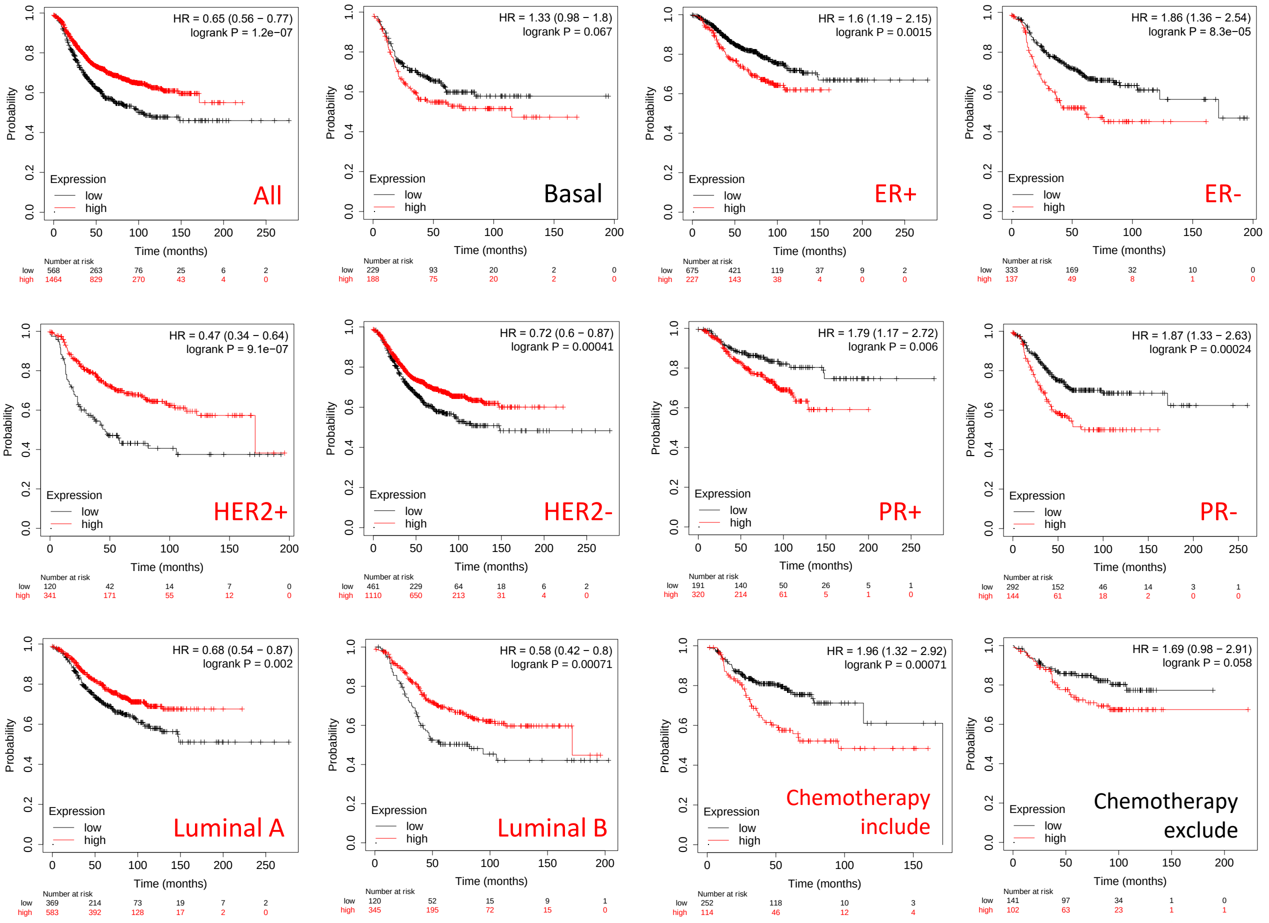

S2-2) GPSM3

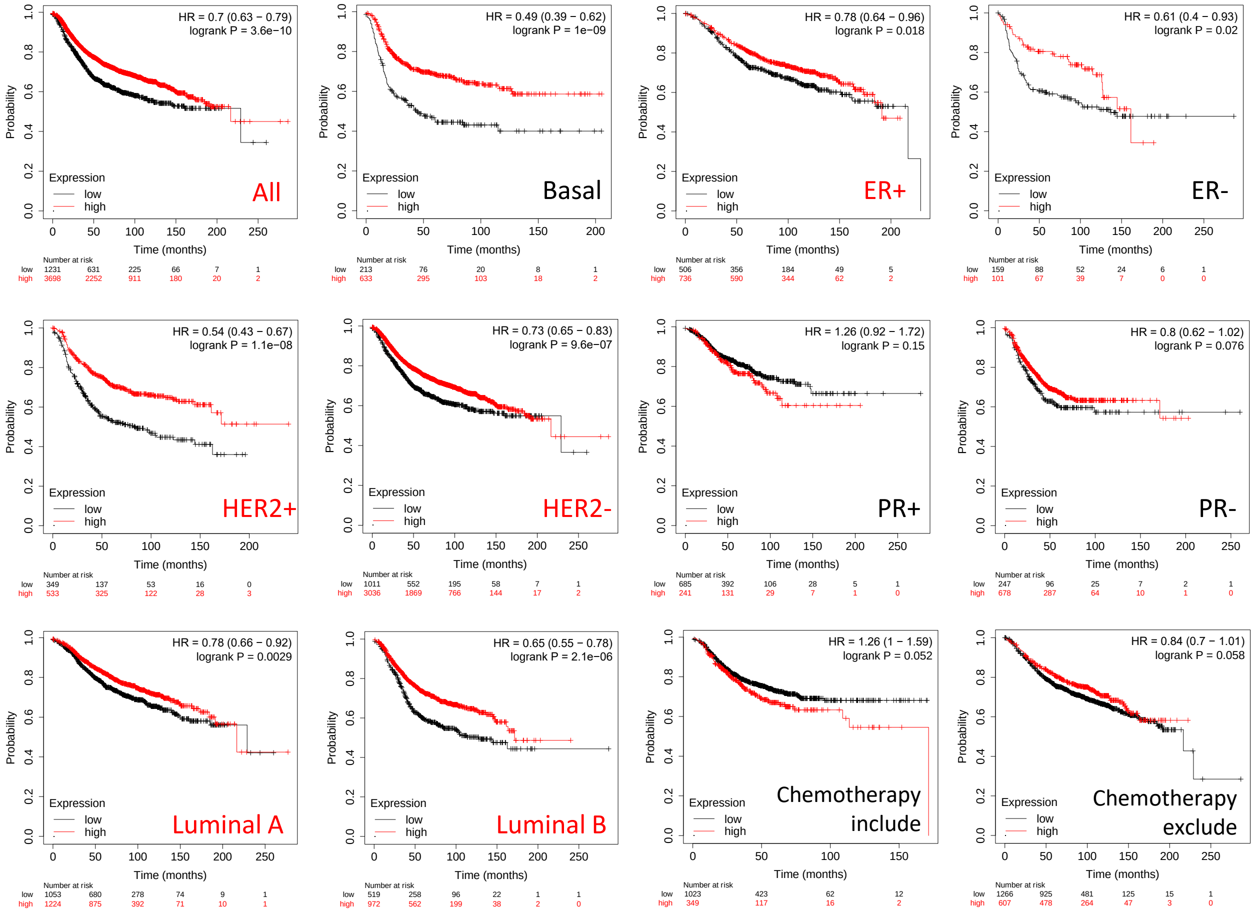
HR = 0.7 (0.63 − 0.79) logrank P = 3.6e−10
All
Number at risk low 1231 631 225 66 7 1
high 3698 2252 911 180 20 2
HR = 0.49 (0.39 − 0.62) logrank P = 1e−09
Basal
Number at risk low 213 76 20 8 1
high 633 295 103 18 2
HR = 0.78 (0.64 − 0.96) logrank P = 0.018
ER+
Number at risk low 506 356 184 49 5
high 736 590 344 62 2
HR = 0.61 (0.4 − 0.93) logrank P = 0.02
ER-
Number at risk low 159 88 52 24 6 1
high 101 67 39 7 0 0
HR = 0.54 (0.43 − 0.67) logrank P = 1.1e−08
HER2+
Number at risk low 349 137 53 16 0
high 533 325 122 28 3
HR = 0.73 (0.65 − 0.83) logrank P = 9.6e−07
HER2-
Number at risk low 1011 552 195 58 7 1
high 3036 1869 766 144 17 2
HR = 1.26 (0.92 − 1.72) logrank P = 0.15
PR+
Number at risk low 685 392 106 28 5 1
high 241 131 29 7 1 0
HR = 0.8 (0.62 − 1.02) logrank P = 0.076
PR-
Number at risk low 247 96 25 7 2 1
high 678 287 64 10 1 0
HR = 0.78 (0.66 − 0.92) logrank P = 0.0029
Luminal A
Number at risk low 1053 680 278 74 9 1
high 1224 875 392 71 10 1
HR = 0.65 (0.55 − 0.78) logrank P = 2.1e−06
Luminal B
Number at risk low 519 258 96 22 1 1
high 972 562 199 38 2 0
HR = 1.26 (1 − 1.59) logrank P = 0.052
Chemotherapy include
Number at risk low 1023 423 62 12
high 349 117 16 2
HR = 0.84 (0.7 − 1.01) logrank P = 0.058
Chemotherapy exclude
Number at risk low 1266 925 481 125 15 1
high 607 478 264 47 3 0
Expression
low
high
Probability
Time (months)

S2-3) GPSM4

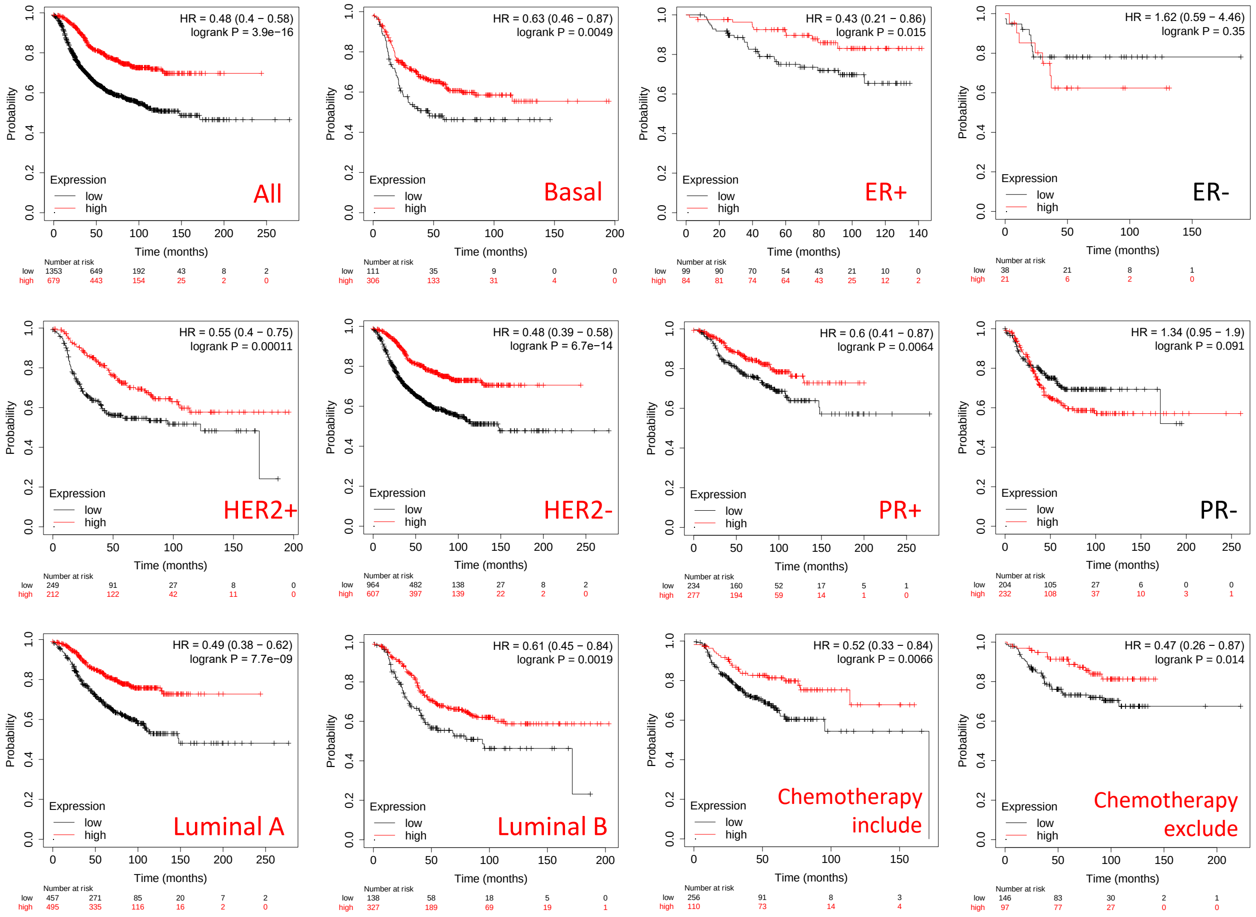
HR = 0.48 (0.4 − 0.58)
logrank P = 3.9e−16
All
Number at risk
low 1353 649 192 43 8 2
high 679 443 154 25 2 0
HR = 0.63 (0.46 − 0.87)
logrank P = 0.0049
Basal
Number at risk
low 111 35 9 0 0
high 306 133 31 4 0
HR = 0.43 (0.21 − 0.86)
logrank P = 0.015
ER+
Number at risk
low 99 90 70 54 43 21 10 0
high 84 81 74 64 43 25 12 2
HR = 1.62 (0.59 − 4.46)
logrank P = 0.35
ER-
Number at risk
low 38 21 8 1
high 21 6 2 0
HR = 0.55 (0.4 − 0.75)
logrank P = 0.00011
HER2+
Number at risk
low 249 91 27 8 0
high 212 122 42 11 0
HR = 0.48 (0.39 − 0.58)
logrank P = 6.7e−14
HER2-
Number at risk
low 964 482 138 27 8 2
high 607 397 139 22 2 0
HR = 0.6 (0.41 − 0.87)
logrank P = 0.0064
PR+
Number at risk
low 234 160 52 17 5 1
high 277 194 59 14 1 0
HR = 1.34 (0.95 − 1.9)
logrank P = 0.091
PR-
Number at risk
low 204 105 27 6 0 0
high 232 108 37 10 3 1
HR = 0.49 (0.38 − 0.62)
logrank P = 7.7e−09
Luminal A
Number at risk
low 457 271 85 20 7 2
high 495 335 116 16 2 0
HR = 0.61 (0.45 − 0.84)
logrank P = 0.0019
Luminal B
Number at risk
low 138 58 18 5 0
high 327 189 69 19 1
HR = 0.52 (0.33 − 0.84)
logrank P = 0.0066
Chemotherapy include
Number at risk
low 256 91 8 3
high 110 73 14 4
HR = 0.47 (0.26 − 0.87)
logrank P = 0.014
Chemotherapy exclude
Number at risk
low 146 83 30 2 1
high 97 77 27 0 0
Expression
low
high
Probability
Time (months)

Supplementary Figure S3. Enrichment pathway analysis of GPSM1, GPSM3 and GPSM4 coexpressed genes in breast cancer database (MetaCore). **A)** Pathway analysis. Potential gene networks and pathways affected by co-expressed genes (right column) and respective log p value (left column). **B)** Biological process analysis. Symbols represent proteins. Arrows depict protein interactions (green, activation; red, inhibition). Thermometer-like histograms indicate microarray gene expression (blue, down-regulation; red, up-regulation)

S3-1. GPSM1

A

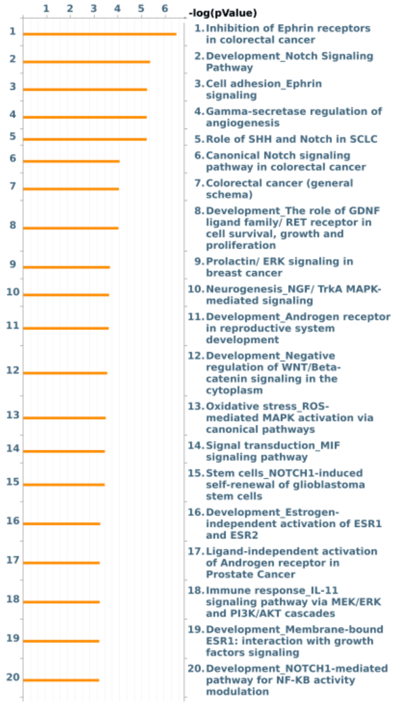

B

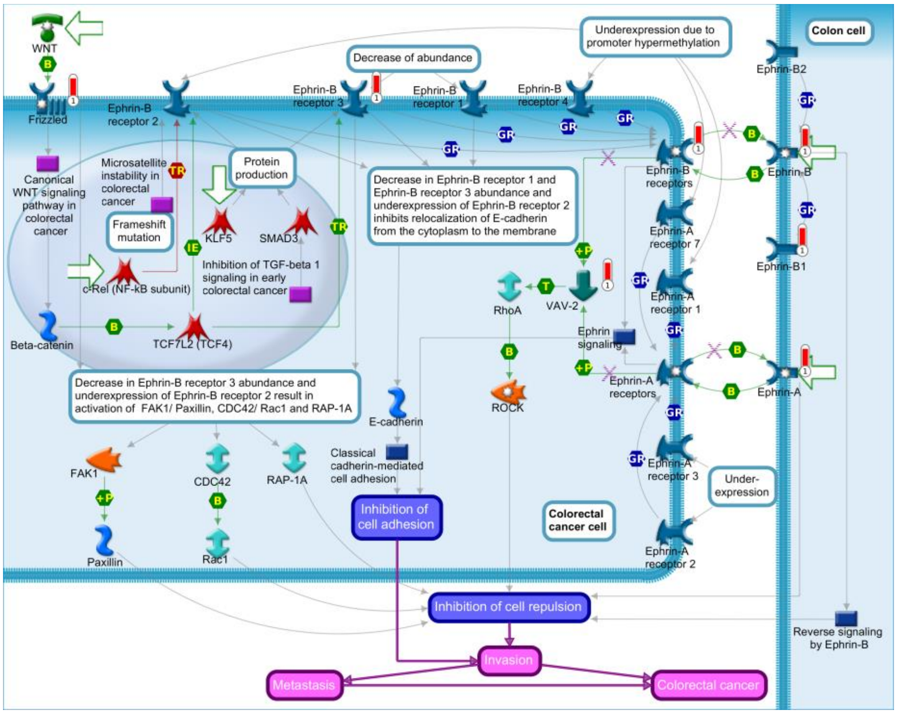

S3-2. GPSM3

A

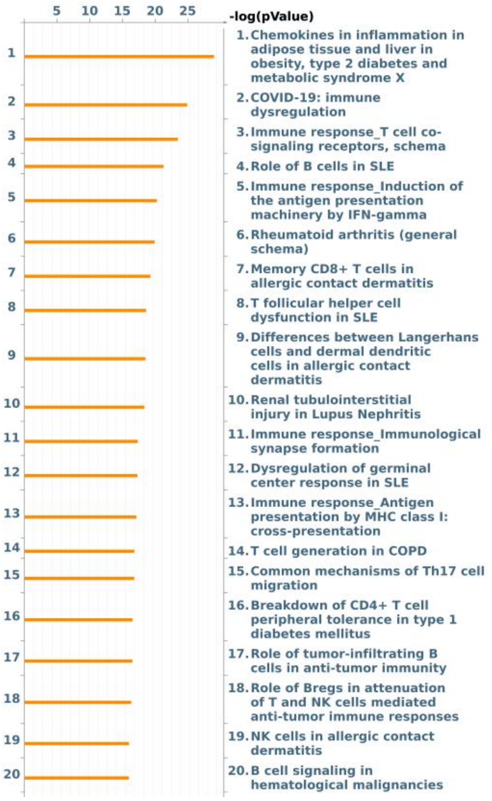

B

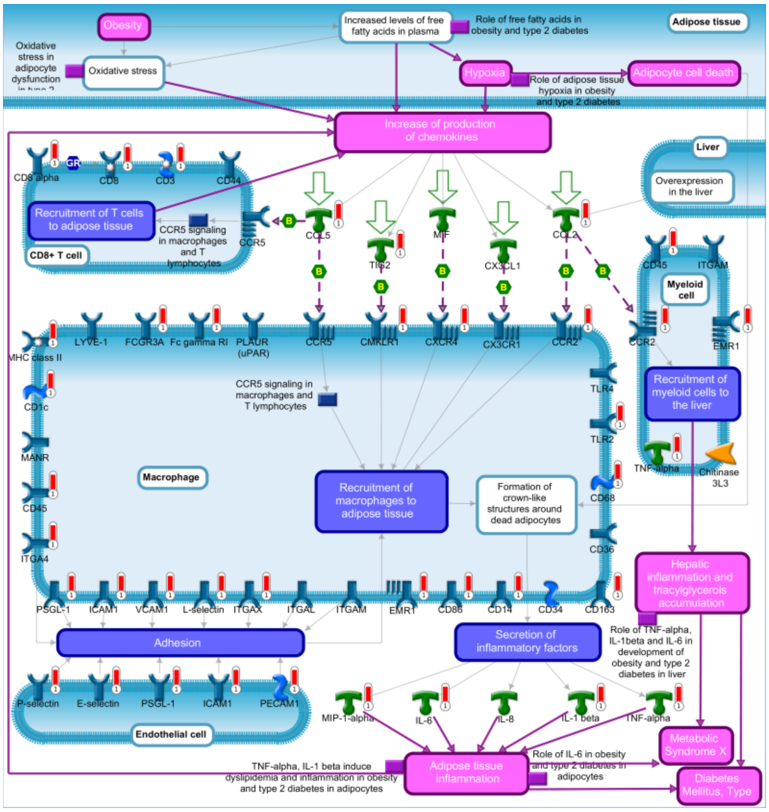

S3-3. GPSM4

A

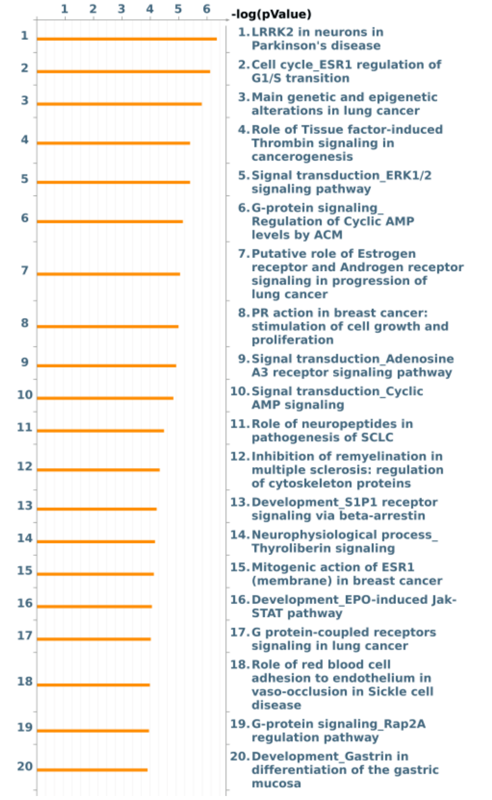

B

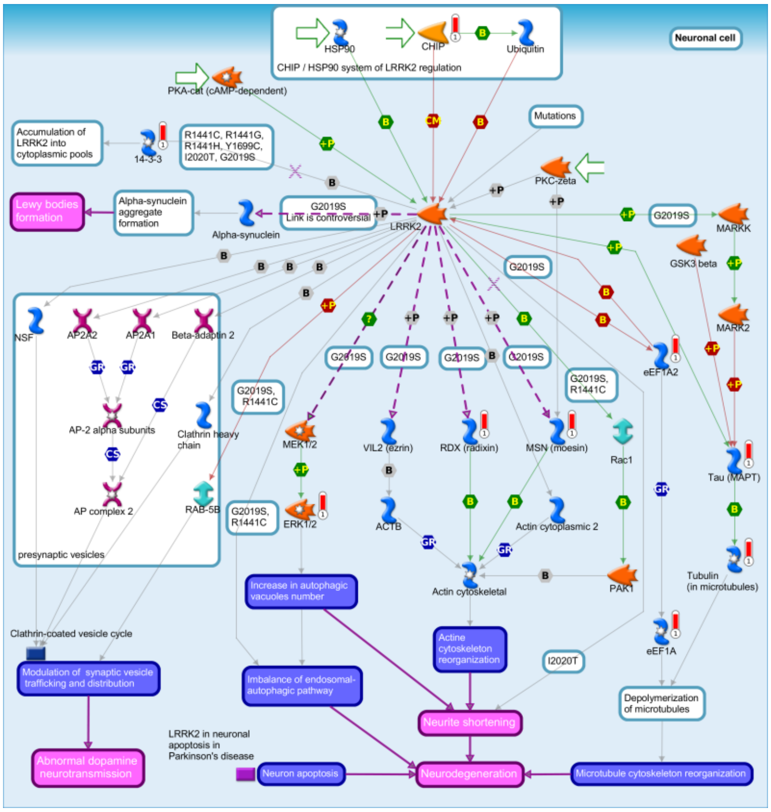

Supplement: Supplementary file 1 [file biomedicines-09-01144-s001.zip › (revised) supplementary.pdf]
